# Enhancing adverse drug reaction reporting through a pharmacy-led multi-component intervention: A real-world pre–post evaluation

**DOI:** 10.1016/j.rcsop.2026.100873

**Published:** 2026-11-02

**Authors:** Musaad Alruwaili, Rana Abu-Huwaij, Mohammad A. Khaleel

**Affiliations:** a College of Pharmacy, Amman Arab University, Amman, Jordan; b Pharmacy Department, Turaif General Hospital, Northern Borders Health Cluster, Health Holding Company, Turaif, Saudi Arabia

**Keywords:** Adverse drug reaction reporting, Pharmacovigilance, QR code, Digital health intervention, Underreporting, Real-world evaluation, Saudi Arabia

## Abstract

**Background:**

Underreporting of adverse drug reactions (ADRs) remains a major challenge in pharmacovigilance despite established reporting systems in Saudi Arabia. Limited healthcare professional (HCP) engagement, inadequate awareness, and low patient participation contribute to low reporting rates, while evidence on integrated real-world interventions remains scarce.

**Objective:**

To evaluate the feasibility and real-world impact of a pragmatic, multi-component intervention combining HCP education, community engagement, and a QR code–based digital ADR reporting system.

**Methods:**

A quasi-experimental pre–post study was conducted at Turaif General Hospital, Saudi Arabia. Seventy-eight matched HCPs completed validated questionnaires before and after the intervention to assess ADR reporting knowledge, perceptions, barriers, and facilitators. The intervention included implementation of a QR code–based reporting system in the outpatient pharmacy, targeted HCP education, and community awareness activities. ADR reports during a 4-month baseline period were compared with those from a 4-month post-intervention period.

**Results:**

HCP knowledge (mean difference = 0.311; *p* = 0.0092) and perceptions (mean difference = 0.354; *p* = 0.0037) improved significantly after the intervention, whereas perceived barriers and facilitators did not change significantly. ADR reports increased from 11 at baseline to 156 post-intervention, representing a 14.2-fold increase. Patients and the public submitted 33% of all reports.

**Conclusion:**

This pragmatic, pharmacy-led intervention was feasible and substantially improved HCP knowledge, perceptions, and ADR reporting. Integrating digital reporting with professional education and community engagement may strengthen patient-centered pharmacovigilance and medication safety.

## Highlights

• A pragmatic, pharmacy-supported, multi-component intervention integrating healthcare provider education, community engagement, and digital reporting was feasible in a real-world hospital setting and improved healthcare providers' knowledge and perceptions of adverse drug reaction reporting.
• Following implementation of the intervention, ADR reporting activity increased 14.2-fold, with approximately one-third of reports originating from patients and members of the public, demonstrating the feasibility and potential value of patient-inclusive approaches in strengthening medication safety surveillance.
• The absence of significant changes in perceived barriers and facilitators indicates that educational interventions should be complemented by organizational and system-level strategies to address persistent challenges in pharmacovigilance practice.

## 1 Introduction

Adverse drug reactions (ADRs) remain a major and largely preventable contributor to patient harm worldwide. Spontaneous reporting systems represent the cornerstone of post-marketing pharmacovigilance, enabling healthcare providers (HCP) and patients to communicate suspected ADRs and support the identification of previously unrecognized safety signals.^1^ However, despite their established value, underreporting remains a persistent global challenge. A median underreporting rate of approximately 94% (interquartile range 82–98%) has been reported across spontaneous reporting systems internationally,^2^ and recent systematic evidence indicates that incomplete reporting continues to compromise the timeliness and effectiveness of drug safety signal detection across healthcare settings.^3^

This challenge is also evident in Saudi Arabia. Engagement with the Saudi Food and Drug Authority's (SFDA) national pharmacovigilance system among frontline HCP remains suboptimal, with only 10% of community pharmacists in the Eastern Province reporting awareness of the national pharmacovigilance center.^4^ Although Saudi Arabia has made substantial investments in digital health transformation under Vision 2030 to enhance healthcare quality and patient safety,^5^ the availability of reporting infrastructure alone may not be sufficient to achieve sustained participation in pharmacovigilance activities.^5^ Pharmacy professionals, as highly accessible HCP and frequent points of contact for medication-related concerns, are strategically positioned to promote ADR detection, reporting, and patient engagement; however, their potential contribution requires adequate knowledge, supportive systems, and practical reporting pathways.

The determinants of ADR underreporting are multifactorial and commonly include knowledge-related, perception-related and structural/logistical barriers.^6^ Saudi-specific studies have similarly identified limited knowledge of reporting procedures, uncertainty regarding reportable events, and practical challenges associated with submission processes as important contributors to poor reporting engagement among HCP.7., 8. These challenges highlight the need for interventions that not only improve awareness but also facilitate convenient and accessible reporting practices within routine healthcare workflows.

Previous interventions have generally targeted individual barriers rather than addressing multiple determinants simultaneously. Educational interventions have demonstrated improvement in HCP’ pharmacovigilance knowledge and, in some settings, increased ADR reporting activity,9., 10. Similarly, digital and mobile-based reporting platforms have improved accessibility and convenience and have been favorably perceived by patients and consumers compared with traditional reporting methods.11., 12. However, evidence remains limited regarding integrated, real-world approaches that combine HCP education, pharmacy-based implementation, community engagement, and simplified digital reporting pathways within routine healthcare settings. In particular, few studies have evaluated whether such multi-component interventions can influence both healthcare provider-related outcomes and actual ADR reporting activity.

This study aimed to address this evidence gap through a pragmatic, real-world evaluation conducted at a general hospital in Saudi Arabia. The objective was to assess the feasibility and impact of a pragmatic, multi-component pharmacovigilance intervention integrating HCP education, community outreach, and QR-code–based digital ADR reporting on HCP’ pharmacovigilance-related knowledge and perception, as well as real-world ADR reporting activity.

## 2 Methods

### 2.1 Study design and setting

A pragmatic, quasi-experimental pre–post evaluation without a control group was conducted at Turaif General Hospital, a 200-bed government hospital serving as the primary public healthcare facility in Turaif Governorate, in the Northern Borders Province, Saudi Arabia. Randomization was not feasible within this real-world healthcare setting; therefore, a pre–post design was selected to evaluate changes in healthcare provider outcomes and ADR reporting activity before and after implementation of the intervention within the same healthcare system.

### 2.2 The multi-component intervention

The intervention consisted of three sequentially implemented components: (1) establishment of a QR code–based digital ADR reporting pathway through the outpatient pharmacy, (2) a structured HCP education program, and (3) a community awareness and engagement campaign. The components were implemented in a staged manner to facilitate integration into routine healthcare workflows and allow evaluation of their combined association with ADR reporting activity.

The digital ADR reporting system was implemented initially through the hospital outpatient pharmacy. ADR reporting activity was monitored during a 4-month baseline period before implementation (June–September 2025) and a 4-month post-implementation period (October 2025–January 2026). Equal-duration observation periods were used to facilitate a direct comparison of ADR reporting activity before and after implementation under routine hospital conditions. The four-month periods were selected to provide comparable observation windows within the study timeframe; however, the duration was not intended to establish the long-term sustainability of the intervention's effects. Approximately 6000 dispensed medications per month were labeled with QR codes linking patients and caregivers to a structured electronic ADR reporting form adapted from the SFDA reporting template. Pharmacy staff provided brief counseling on the reporting process at the point of dispensing. Submitted reports were reviewed by the hospital pharmacovigilance officer and subsequently forwarded to the SFDA.

Following implementation of the digital reporting pathway, a structured HCP education program and community outreach campaign were conducted during 27 November–12 December 2025, within the post-implementation observation period. The HCP education component included a live online educational session, a 13-unit self-paced pharmacovigilance course hosted on the hospital website (available from 20 October to 20 December 2025), and supporting educational materials distributed through digital channels, including videos, posters, podcasts, and electronic booklets.

The community outreach component aimed to increase public awareness of ADR reporting and promote patient participation in pharmacovigilance. This component was included because patients and other medication users are directly exposed to medicines and may be the first to recognize suspected adverse drug reactions. Engaging the public in pharmacovigilance is therefore complementary to educating HCPs and establishing accessible reporting pathways within healthcare settings.

Approximately 1500 members of the public were reached through activities conducted at selected community locations in Turaif, including a shopping center, public park, and walking track, all located within the hospital's surrounding community**.** Trained volunteers delivered educational messages using printed materials, digital posters, and awareness resources. These activities were intended to promote awareness of medication safety and encourage the reporting of suspected ADRs, rather than to directly evaluate the hospital-based QR reporting system. Accordingly, the community outreach campaign and the hospital-based reporting intervention addressed complementary aspects of ADR reporting but targeted different populations and settings. The campaign was registered through the Saudi Ministry of Health volunteering platform (Reference No. MOHEV97161).

### 2.3 Participants and eligibility

The required sample size for healthcare providers (HCPs) was calculated using Cochran's formula with finite population correction, appropriate for a defined population of eligible hospital staff. The total eligible HCP population (*N* = 159) included all full-time physicians, pharmacists, and assistant pharmacists employed at Turaif General Hospital during the study period. Assuming a 95% confidence level (Z = 1.96), a 5% margin of error (e = 0.05), and an anticipated prevalence (p) of adequate baseline pharmacovigilance awareness of 11% based on regional evidence of limited awareness among HCP [4], the initial sample size (n₀) was estimated as:

$$n₀=Z^{²}p\frac{\left(1-p\right)}{e^{²}}=150$$

After applying the finite population correction for the 159 eligible providers, the required sample size (n) was reduced to 78 participants:

$$n=\frac{n₀}{\left[1+\frac{n₀-1}{N}\right]}=78$$

Participants were recruited using non-probability convenience sampling. Healthcare providers who were on leave, belonged to other professional categories, or declined participation were excluded.

For the community outreach component, eligible participants were adults aged ≥18 years who agreed to participate and were able to communicate effectively in Arabic or English. Individuals unable to provide informed consent were excluded.

For the QR code–based ADR reporting component, eligible users were adult outpatients (≥18 years) who received medications labeled with QR codes and had access to a smartphone to complete the electronic reporting form. Individuals unable to access or use digital reporting platforms because of technical or other limitations were excluded.

### 2.4 Data collection instrument

A structured questionnaire was developed based on a review of the pharmacovigilance literature and previous studies assessing HCP’ engagement with adverse drug reaction (ADR) reporting. The questionnaire consisted of two sections. The first section collected participants' demographic characteristics, including gender, age, years of professional experience, clinical area/specialty, and professional level. The second section assessed four pharmacovigilance-related constructs: Knowledge (4 items), Perception (3 items), Barriers (8 items), and Facilitators (6 items). All items were rated using a 5-point Likert scale.

The Knowledge construct assessed awareness of ADR reporting requirements and included statements that were inconsistent with Saudi pharmacovigilance reporting requirements (e.g., the belief that only high-risk medicines require ADR reporting). Agreement with these statements indicated lower awareness; therefore, items were reverse-scored, with higher scores reflecting more accurate pharmacovigilance knowledge. The Perception construct evaluated participants' views regarding the effectiveness, accessibility, and patient safety value of ADR reporting systems. The Barriers construct assessed the extent to which specific factors hinder ADR reporting, with higher scores indicating greater perceived barriers. The Facilitators construct assessed the extent to which specific organizational and practical supports could encourage ADR reporting.

Before analysis, a predefined measurement model was established for each construct. Perception and Facilitators were considered reflective constructs, where items are expected to measure a common underlying concept; therefore, internal consistency was assessed using Cronbach's alpha with Feldt confidence intervals. In contrast, Knowledge and Barriers were considered formative indices because their items represent distinct and non-interchangeable domains rather than manifestations of a single underlying construct. Accordingly, these constructs were interpreted at the item level, and internal consistency coefficients were not used as indicators of scale reliability.

Content validity was established through review by pharmacovigilance and clinical pharmacy experts.

A pilot study involving 20 healthcare professionals (HCPs), who were excluded from the final analysis, was conducted to assess the clarity, comprehensibility, and feasibility of questionnaire administration. Participants completed and reviewed the questionnaire to identify ambiguous wording, comprehension difficulties, and practical administration issues. The questionnaire was initially developed in English, translated into Arabic, and reviewed to ensure semantic equivalence between the two versions. The sample size was guided by the pilot's feasibility objectives rather than a formal statistical power calculation, consistent with recommendations for pilot studies.13., 14.

### 2.5 ADR reporting data collection

ADR reporting activity was assessed during two predefined evaluation periods: a 4-month baseline period (June–September 2025), during which reports were submitted through the hospital's existing SFDA reporting pathway, and a 4-month post-implementation period (October 2025–January 2026), following introduction of the QR code–based electronic reporting system through the “Tiqath” platform. Each submitted report was assigned a unique reference identifier to facilitate data tracking, review, and prevention of duplicate entries.

All ADR reports received during both periods were reviewed. Reports were classified according to ADR severity using the Hartwig and Siegel scale.^15^ Extracted variables included the suspected medication, reporting source (healthcare provider or patient/public), and relevant clinical characteristics available within the reporting system. ADR reporting activity was evaluated by comparing the number and characteristics of reports submitted during the baseline and post-implementation periods.

### 2.6 Statistical analysis

Distributional normality was assessed for each construct at both assessment points using the Shapiro–Wilk test. Changes in Knowledge, Perception, Barriers, and Facilitators scores between pre- and post-intervention assessments were evaluated using the Wilcoxon signed-rank test. A paired *t*-test was additionally performed as a sensitivity analysis for constructs where the distribution of paired differences met normality assumptions.

For each construct, the matched-pairs rank-biserial correlation was reported as the effect size for the Wilcoxon signed-rank test, together with Cohen's *d* for paired samples to provide an additional standardized estimate of change magnitude. The rank-biserial correlation was interpreted as the non-parametric effect size corresponding to the direction and magnitude of paired differences.

Multiple testing across the four primary constructs was controlled using the Benjamini–Hochberg false discovery rate (FDR) correction. The same procedure was applied to item-level comparisons within each construct.

Baseline differences according to gender were assessed using the Mann–Whitney *U* test with rank-biserial correlation as the effect size. Differences across professional categories were evaluated using the Kruskal–Wallis test with epsilon squared as the effect size. Associations between continuous demographic variables (age and years of professional experience) and construct scores were examined using Spearman's rank correlation coefficient.

Changes in ADR reporting volume were assessed by comparing the number of reports submitted during the 4-month baseline and 4-month post-implementation periods using an exact Poisson rate-ratio test, with results expressed as a rate ratio (RR) and 95% confidence interval.

All statistical tests were two-sided, and statistical significance was set at *p* < 0.05. Analyses were performed using R version 4.3.3 (R Core Team, Vienna, Austria).

### 2.7 Ethical considerations

The study protocol was approved by the Institutional Review Board in Arar, Northern Borders Health Cluster (IRB Log No. N1C-IRB-26-02-06), prior to implementation. Written informed consent was obtained from all HCP participants, with consent confirmed as the first questionnaire item. Participation in the community outreach campaign and submission of ADR reports via the QR-code system were considered voluntary and were conducted without collection of identifiable personal data.

## 3 Results

### 3.1 Participant characteristics

A total of 159 eligible HCPs were invited to participate at Turaif General Hospital. Of these, 78 completed both the pre- and post-intervention questionnaires, yielding a response rate of 49.1%. Table 1 presents the demographic characteristics of HCP participants. The majority of participants were male (*n* = 55, 70.5%), while 23 (29.5%) were female. The mean age was 42.4 years (SD 6.8; range 30–58 years), and the mean duration of professional experience was 14.0 years (SD 6.6). Pharmacy staff comprised 29.5% (*n* = 23) of the sample, with the remainder representing a range of physician specialties, most commonly pediatrics and general practice (11 each, 14.1%). By professional level, specialists constituted the largest group (*n* = 29, 37.2%), followed by pharmacy technicians (*n* = 15, 19.2%), residents (*n* = 15, 19.2%), consultants (*n* = 11, 14.1%), and pharmacists (*n* = 8, 10.3%).

**Table 1:** Demographic Characteristics of Participating Healthcare Providers (*N* = 78).

Characteristic	Category	n (%) or Mean ± SD
Gender	Male	55 (70.5%)
	Female	23 (29.5%)
Age (years)	–	42.40 ± 6.80
Years of Experience	–	14.00 ± 6.60
Clinical area / specialty	Pharmacy	23 (29.5%)
	Pediatrics	11 (14.1%)
	General Practice	11 (14.1%)
	Other physician specialties⁎	33 (42.3%)
Professional Level	Specialist	29 (37.2%)
	Pharmacy Technician	15 (19.2%)
	Resident	15 (19.2%)
	Consultant	11 (14.1%)
	Pharmacist	8 (10.3%)

*Abbreviations: SD, standard deviation; N, total sample size; n, subgroup count.*

*Age range: 30–58 years. Percentages are calculated based on the total sample (N* *=* *78).*

⁎ *Includes individual physician specialties each represented by fewer than 12 participants (OB/GYN, general surgery, ENT, internal medicine, radiology, urology, anesthesiology, orthopedic surgery, cardiology, ophthalmology, dermatology, emergency medicine, and family medicine), combined for conciseness; full specialty-level breakdown is provided in the Supplementary Material.*

### 3.2 Instrument reliability and measurement model

Table 2 presents the measurement characteristics of the questionnaire constructs. The two reflective constructs demonstrated excellent internal consistency at both assessment points, with Cronbach's alpha increasing from 0.893 (95% CI: 0.844–0.929) to 0.934 (95% CI: 0.904–0.956) for Perception and from 0.938 (95% CI: 0.914–0.957) to 0.973 (95% CI: 0.963–0.981) for Facilitators. The Knowledge construct, specified as a formative index, showed descriptive Cronbach's alpha values of 0.629 (95% CI: 0.474–0.747) at baseline and 0.806 (95% CI: 0.725–0.868) following the intervention.

**Table 2:** Measurement model and internal consistency (Cronbach's alpha, Feldt 95% CI) by construct.

Construct	Model	Items	α pre (95% CI)	α post (95% CI)
Knowledge	Formative index	4	0.629 (0.474–0.747)	0.806 (0.725–0.868)
Perception	Reflective scale	3	0.893 (0.844–0.929)	0.934 (0.904–0.956)
Barriers	Formative index	8	Not applicable⁎	Not applicable⁎
Facilitators	Reflective scale	6	0.938 (0.914–0.957)	0.973 (0.963–0.981)

⁎ *The eight barrier items are a formative index; the composite alpha is negative and the mean inter-item correlation is near zero, so a single reliability coefficient is not meaningful. Item-level results are in Supplementary Table S1. Cronbach's alpha was not calculated for the **Barriers** construct because it was specified* a priori *as a formative index comprising distinct, non-interchangeable items. Internal consistency is not an appropriate reliability measure for formative constructs; therefore, barrier items were analyzed individually.*

Because the Barriers construct was specified as a formative index composed of distinct and non-interchangeable items, internal consistency was not considered an appropriate measure of reliability and was therefore not calculated. Consistent with the predefined measurement model, barrier items were analyzed and interpreted individually rather than as a composite reflective scale.

### 3.3 Pre- versus post-intervention construct outcomes

Table 3 summarizes changes in the four study constructs following the intervention. Significant improvements were observed in healthcare providers' Knowledge and Perception of ADR reporting. Mean Knowledge scores increased from 3.90 (SD 0.75) to 4.21 (SD 0.81) (V = 748.5, *p* = 0.009; Benjamini–Hochberg-adjusted *p* = 0.019), corresponding to a small-to-moderate effect size (rank-biserial correlation = 0.362; Cohen's *d* = 0.309). Similarly, Perception scores increased from 3.90 (SD 0.97) to 4.26 (SD 0.90) (V = 501.5, *p* = 0.004; adjusted *p* = 0.015), with a moderate effect size (rank-biserial correlation = 0.433; Cohen's *d* = 0.278).

**Table 3:** Pre- and post-intervention changes in questionnaire construct scores (Wilcoxon signed-rank test, *N* = 78).

Construct	Pre mean (SD)	Post mean (SD)	Diff (95% CI)	p	p (BH)	r (rank-biserial) (95% CI)	d (95% CI)
**Knowledge**	3.90 (0.75)	4.21 (0.81)	0.31 (0.08 to 0.54)	0.009	0.019	0.362 (0.088 to 0.593)	0.309 (0.080 to 0.535)
**Perception**	3.90 (0.97)	4.26 (0.90)	0.35 (0.07 to 0.64)	0.004	0.015	0.433 (0.120 to 0.671)	0.278 (0.051 to 0.503)
**Barriers**	3.19 (0.34)	3.17 (0.38)	−0.01 (−0.12 to 0.09)	0.702	0.702	−0.055 (−0.342 to 0.226)	−0.028 (−0.250 to 0.194)
**Facilitators**	4.02 (0.81)	4.09 (0.92)	0.06 (−0.19 to 0.31)	0.244	0.326	0.173 (−0.131 to 0.445)	0.056 (−0.166 to 0.278)

V, Wilcoxon signed-rank statistic (sum of positive-signed ranks); r, matched-pairs rank-biserial correlation; d, Cohen's d for paired data; BH, Benjamini-Hochberg adjusted p across the four constructs.

No statistically significant changes were observed in the Barriers or Facilitators constructs following the intervention. **Although an overall descriptive mean is presented for the Barriers construct, interpretation focused primarily on the individual barrier items because the construct was specified a priori as a formative index comprising distinct and non-interchangeable barriers.** The mean Barriers score changed minimally from 3.19 (SD 0.34) to 3.17 (SD 0.38) (*p* = 0.702), while the mean Facilitators score increased slightly from 4.02 (SD 0.81) to 4.09 (SD 0.92) (*p* = 0.244). These findings remained unchanged after adjustment for multiple comparisons.

Sensitivity analyses using paired *t*-tests for constructs in which the paired differences met normality assumptions yielded results consistent with the primary Wilcoxon signed-rank analyses.

### 3.4 Item-level and subgroup findings

Item-level analyses provided further insight into the observed changes in Knowledge, Perception, and Barriers (Supplementary Table S1). Within the Perception construct, improvements were primarily driven by two items: agreement that the ADR reporting system was clear for healthcare providers increased by 0.51 points (*p* = 0.002; adjusted *p* = 0.005), and perceived accessibility of the system increased by 0.42 points (*p* = 0.004; adjusted *p* = 0.006). The item related to the importance of ADR reporting for patient safety showed no significant change, likely due to high baseline scores.

Among the Knowledge items, the greatest improvement was observed for the misconception that only serious ADRs require reporting (mean difference = 0.47; adjusted *p* = 0.043), indicating improved recognition that a broader range of suspected ADRs should be reported.

Because the Barriers construct was specified as a formative index, interpretation focused on individual items rather than an overall composite score. Only one barrier item demonstrated a significant change: the perception that an ADR was too unimportant to report decreased from 3.58 to 3.04 (mean difference = −0.54; *p* = 0.003; adjusted *p* = 0.022; rank-biserial correlation = −0.49). Other structural and organizational barriers, including workload, lack of convenient reporting systems, insufficient feedback, and lack of incentives, showed no significant changes following the intervention.

Baseline subgroup analyses showed that female participants had higher Knowledge scores than male participants (mean 4.20 vs. 3.77; U = 838.0; *p* = 0.024); however, no significant gender differences were observed for Perception, Barriers, or Facilitators. After adjustment for multiple comparisons, construct scores did not differ significantly across professional categories. Spearman correlation analyses showed no significant associations between construct scores and age or years of professional experience (all adjusted *p* > 0.05). Detailed subgroup analyses are presented in Supplementary Tables S2–S4.

### 3.5 Implementation of the educational and community components

Implementation indicators demonstrated substantial reach and engagement with the educational and community components of the intervention. The live educational lecture was attended by 25 of 39 eligible pharmacists (64%), and 90% of attendees achieved full marks on the 10-item post-lecture knowledge assessment. The self-paced online pharmacovigilance course enrolled 186 healthcare providers, with 162 participants (87%) completing all course units.

The community outreach component involved 25 trained volunteers and reached approximately 1500 members of the public through awareness activities conducted at selected community locations. These findings indicate successful delivery and uptake of the intervention components among both healthcare providers and the community.

### 3.6 Change in ADR reporting activity following implementation of the multi-component intervention”

During the baseline period, 11 ADR reports were submitted through the hospital's existing reporting pathway (mean 2.75 per month; range 2–4). Following implementation of the multi-component intervention, 156 reports were received (mean 39.0 per month; range 19–52), corresponding to a 14.2-fold increase in the reporting rate (RR 14.2, 95% CI 7.7–29.0; *p* < 0.001). Monthly reporting rose progressively throughout the post-implementation period (19, 37, 48 and 52 reports), whereas baseline counts were stable (Fig. 1).

**Fig. 1:**
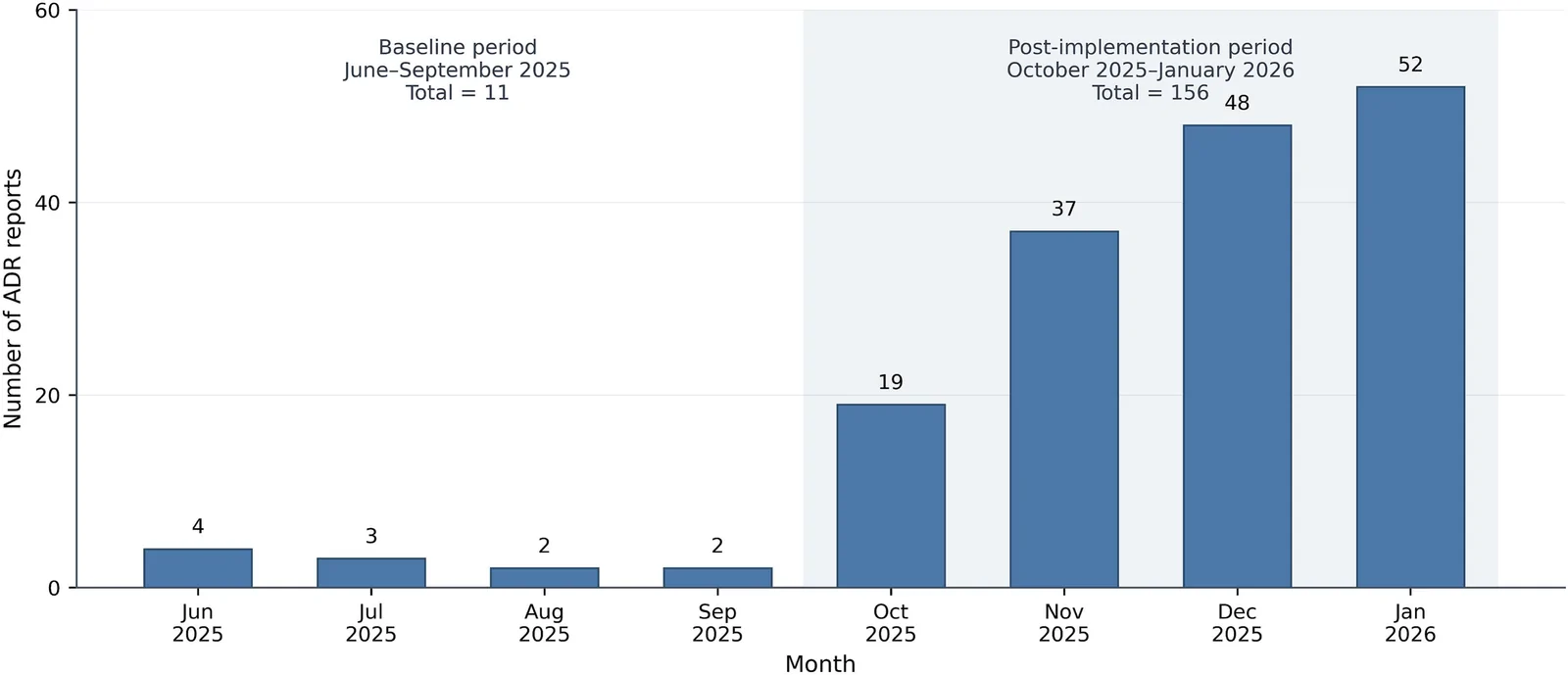
Monthly ADR reporting activity during the baseline and post-implementation periods.

Among reports submitted during the post-implementation period, approximately one-third (33%) originated from patients and members of the public, demonstrating meaningful engagement of non-healthcare provider reporters in ADR surveillance. The increase in reporting activity following implementation suggests improved accessibility and engagement with ADR reporting processes within the hospital and surrounding community.

### 3.7 Severity and characteristics of QR-code–derived ADR reports

Fig. 2 summarizes the severity and clinical characteristics of the 156 ADR reports submitted through the QR code–based reporting pathway during the post-implementation period. Most reports were classified as mild (54.8%), followed by moderate (29.8%) and severe (15.4%) reactions according to the Hartwig and Siegel severity scale.

**Fig. 2:**
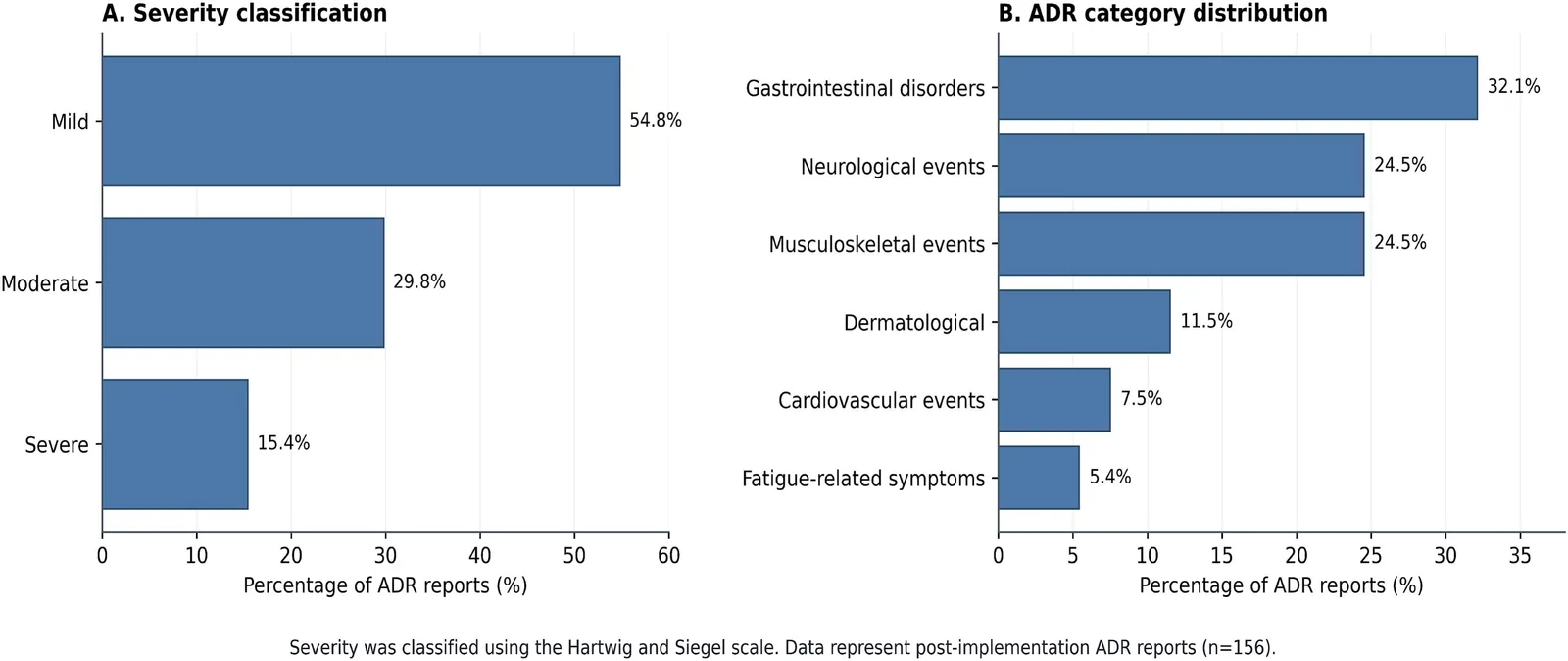
Severity (A) and category (B) distribution of ADR Reports submitted through the QR code–based reporting pathway during the post-implementation period**.**

Paracetamol was the most frequently suspected medication, accounting for 15.38% of reports, followed by amlodipine (14.42%). Oral administration represented the predominant route of administration, while topical, intramuscular, inhalation, and other routes were reported less frequently.

Regarding reported clinical manifestations, gastrointestinal disorders were the most commonly reported ADR category (32.1%), followed by neurological and musculoskeletal disorders (24.5% each). Moreover, the dashboard identified 39 reports involving sensitive age groups and generated alerts for five pregnancy-related and two lactation-related cases. It also characterized medication-source patterns, including 79 reports involving medications obtained from the hospital and 53 involving medications obtained from community pharmacies.

## 4 Discussion

This study evaluated the implementation of a pragmatic, multi-component pharmacovigilance intervention combining a QR-code–based digital ADR reporting pathway, healthcare provider education, and community engagement activities within a real-world hospital setting in Saudi Arabia. The intervention was associated with significant improvements in healthcare providers' knowledge and perception regarding ADR reporting and coincided with a substantial increase in ADR reporting activity during the post-implementation period. Importantly, approximately one-third of reports submitted after implementation originated from patients and members of the public, demonstrating meaningful expansion of participation beyond healthcare professionals. These findings provide evidence of successful implementation of the intervention and indicate changes at both the healthcare provider attitudinal level and the pharmacovigilance reporting system level.

The observed improvements in Knowledge and Perception are consistent with previous educational interventions demonstrating that structured pharmacovigilance training can enhance healthcare providers' awareness and understanding of ADR reporting requirements.9., 10., 16.

In the present study, improvements remained statistically significant after adjustment for multiple comparisons, suggesting that the observed changes were unlikely to be attributable to multiple testing. However, because the educational intervention was implemented as part of a broader multi-component strategy and the study lacked a control group, the observed improvements should be interpreted as associations with the intervention rather than definitive causal effects.

Knowledge scores rose by 0.31 points (95% CI 0.08–0.54) and perception scores by 0.35 points (95% CI 0.07–0.64). These gains reached statistical significance, but the standardized mean differences were small (Cohen's d ≈ 0.3) and the rank-biserial correlations moderate (*r* = 0.36–0.43), and the lower confidence limits allow for a small true improvement. No established minimal clinically important difference exists for these study-specific constructs, so the score differences alone cannot show whether the changes are clinically or practically meaningful. The rise in ADR reporting during the same period provides a complementary implementation outcome, but our design cannot link individual score changes to reporting behavior. Future studies should define meaningful change thresholds for pharmacovigilance knowledge and perception measures, preferably with anchor-based methods supported by distribution-based analyses, and test whether score changes predict sustained reporting and the quality and completeness of ADR reports.

The magnitude of this increase is broadly consistent with prior evidence that structured pharmacovigilance interventions can be associated with substantial gains in reporting activity,^17^ as previously observed in a Jordanian educational intervention in which reporting volume rose from 546 to 1060 submissions following implementation.^10^ The observed rise in reporting activity also coincided with implementation of the multi-component intervention, which incorporated a digital QR-code–based reporting channel, aligning with prior findings that digital tools are associated with improved reporting engagement and are preferred by patients and consumers over traditional reporting methods.11., 12. In addition, baseline reports were captured through the hospital's existing reporting route, whereas post-implementation reports were captured through the newly introduced QR-code form, so part of the observed increase reflects the availability of a new, low-friction reporting channel rather than a change in reporting behavior alone. However, because the QR-code system, healthcare provider education, and community outreach were implemented as a coordinated, staged multi-component intervention rather than as isolated arms, the relative contribution of each individual component to the observed increase in reporting activity cannot be determined from the present study. The overall pattern nonetheless suggests that coordinated, multi-component pharmacovigilance strategies may enhance reporting engagement when implemented under routine clinical practice.

The contribution of patients and members of the public represents an important finding of this intervention, as approximately one-third of post-implementation ADR reports originated from non-healthcare provider reporters. This finding is in agreement with the existing literature suggesting that patient-submitted reports can be a valuable and supplemental source of pharmacovigilance data, capturing medication-related experiences that may not be consistently documented by healthcare providers.^18^ This finding suggests that accessible reporting systems could facilitate broader participation by patients and members of the public alongside healthcare professionals in pharmacovigilance reporting.^19^

The digital reporting component also provided operational advantages beyond increasing accessibility to ADR reporting. The Tiqath platform enabled standardized data capture aligned with the SFDA reporting template, generation of unique report identifiers, structured tracking of report status, and organized review of submitted cases by the hospital pharmacovigilance team. The platform also facilitated identification of clinically relevant characteristics, including reports involving clinically important patient groups, including pregnancy, lactation, and different age categories. These functions illustrate the potential value of integrating digital reporting tools with pharmacovigilance workflows to support systematic review, follow-up, and safety surveillance in routine healthcare environments. Nevertheless, because the digital pathway was introduced alongside healthcare provider education and community outreach, its independent contribution to the increase in reporting activity cannot be determined.

Despite improvements in Knowledge and Perception, no significant changes were observed in the Barriers or Facilitators constructs. This finding suggests that structural and organizational factors affecting ADR reporting may be less amenable to short-term educational and awareness interventions. Because the Barriers construct was predefined as a formative index consisting of distinct obstacles, interpretation focused on individual items rather than a composite score. At the item level, only the perception that an ADR was too unimportant to report significantly decreased after the intervention, whereas other barriers, including workload, lack of feedback, limited reporting convenience, and lack of incentives, remained unchanged. This pattern is consistent with previous literature indicating that many ADR reporting barriers are embedded within institutional workflows and organizational processes and may require targeted system-level interventions rather than education alone.8., 20. Similarly, the absence of significant change in Facilitators may partly reflect high baseline facilitator scores, leaving limited potential for measurable improvement after intervention.

A major strength of this study is the evaluation of both attitudinal outcomes and actual reporting behavior within routine clinical practice. Unlike studies assessing only knowledge or attitudes, this evaluation examined changes in healthcare provider perceptions alongside real-world ADR reporting activity. The pragmatic design allowed assessment of the intervention under usual hospital conditions rather than an artificial research environment. In addition, the inclusion of patient and public reporting expanded the assessment beyond healthcare professionals alone and demonstrated the feasibility of engaging multiple reporting sources. The construct-specific measurement approach also strengthened interpretation by avoiding inappropriate use of internal consistency metrics for formative indices.

Several limitations should be considered when interpreting these findings. First, the quasi-experimental pre–post design without a control group limits the ability to establish causal relationships between the intervention and observed changes. Although the findings demonstrate temporal associations between implementation of the intervention and changes in reporting activity, the contribution of external factors or concurrent changes in pharmacovigilance practice cannot be completely excluded. Future studies using controlled or stepped-wedge designs could provide stronger evidence regarding intervention effectiveness.

Second, healthcare providers were recruited using non-probability convenience sampling, which may limit the representativeness of the sample and introduce selection bias.

Third, because the intervention consisted of multiple coordinated components, including implementation of the QR-code reporting pathway, healthcare provider education, and community outreach, the independent contribution of each component could not be determined. In addition, baseline and post-implementation ADR reports were captured through different reporting pathways, meaning that the observed increase in reporting activity may reflect both improved accessibility of the new reporting system and changes in reporting behavior following the intervention. Therefore, the findings should be interpreted as an association with the overall intervention rather than as a direct effect of any single component. Future evaluations using controlled staggered implementation or component-specific analyses may help identify the relative contribution of individual intervention elements. The introduction of a new reporting channel may have contributed to increased accessibility and engagement, and a potential novelty or Hawthorne effect cannot be excluded, as increased attention to ADR reporting during the implementation period may have temporarily influenced reporting behavior. Longer follow-up periods are needed to determine whether the observed increase can be sustained over time.

Finally, this study was conducted in a single hospital setting, which may limit generalizability to other healthcare systems with different organizational structures, resources, or reporting cultures. Nevertheless, the intervention was designed as a pragmatic, low-cost model that could potentially be adapted to similar healthcare environments seeking to strengthen pharmacovigilance participation.

Overall, the findings suggest that an integrated pharmacovigilance strategy combining accessible digital reporting, healthcare provider education, and community engagement can enhance participation in ADR reporting within routine clinical practice. However, the persistence of organizational and structural barriers indicates that educational approaches alone may be insufficient to achieve comprehensive and sustainable improvements. Future pharmacovigilance strengthening initiatives should combine capacity building with system-level strategies, including improved feedback mechanisms, workflow integration, and institutional support for reporting.

The broader implications of these findings align with the World Health Organization's Smart Pharmacovigilance Strategy**,** which emphasizes adaptive, risk-based, digitally enabled, and sustainable safety systems integrated within broader health infrastructures.^21^ By combining stakeholder engagement, digital innovation, and capacity building, pragmatic interventions such as the present model may contribute to strengthening medicine safety surveillance and supporting more responsive pharmacovigilance systems.

## 5 Conclusion

This pragmatic, real-world evaluation demonstrates the feasibility of implementing a multi-component pharmacovigilance intervention combining healthcare provider education, community engagement, and a digital ADR reporting pathway within routine hospital practice. The intervention was associated with improved healthcare provider knowledge and perception regarding ADR reporting and with a substantial increase in ADR reporting activity, including meaningful contributions from patients and members of the public.

However, the lack of significant changes in perceived barriers and facilitators highlights that educational and awareness-based strategies alone may not be sufficient to overcome persistent organizational and system-level challenges. Sustainable improvement in pharmacovigilance practice will likely require complementary institutional strategies, including workflow integration, feedback mechanisms, and continued support for reporting processes.

These findings provide practical insights for healthcare systems seeking to strengthen pharmacovigilance participation through patient-inclusive and digitally enabled approaches. Future controlled and longer-term evaluations are warranted to determine the sustainability and broader applicability of such interventions.

## CRediT authorship contribution statement

**Musaad Alruwaili:** Writing – original draft, Methodology, Formal analysis, Data curation, Investigation. **Rana Abu-Huwaij:** Writing – review & editing, Supervision, Conceptualization, Project administration, Formal analysis. **Mohammad A. Khaleel:** Writing – review & editing, Validation, Formal analysis.

## Consent to participate

Written informed consent was obtained from all healthcare providers who participated in the questionnaire-based evaluation. Participation in the community outreach activities and submission of adverse drug reaction reports through the QR-code–based reporting system were voluntary. No personally identifiable information was collected through the reporting pathway.

## Ethics approval

The study was approved by the Institutional Review Board of Arar, Northern Borders Health Cluster (IRB Log No. N1C-IRB-26-02-06). The study was conducted in accordance with institutional ethical requirements and applicable regulations.

## Funding

This research received no external funding.

## Declaration of competing interest

The authors declare that they have no known competing financial interests or personal relationships that could have appeared to influence the work reported in this manuscript.

## Data Availability

The datasets generated and/or analyzed during the current study are available from the corresponding author upon reasonable request.
