# Supplementary material for: Enhancing adverse drug reaction reporting through a pharmacy-led multi-component intervention: A real-world pre–post evaluation
Source: Explor Res Clin Soc Pharm. 2026 Nov 2;24:100873. doi: 10.1016/j.rcsop.2026.100873 (PMC13756347; doi:10.1016/j.rcsop.2026.100873)
Supplement: Supplementary file 1 [file mmc1.docx]

# **Supplementary Materials**

**Supplementary Table S1. Pre- and Post-Intervention Comparison of Individual Questionnaire Items (Wilcoxon Signed-Rank Test, N=78)**

| **Construct** | **Item** | **Pre** | **Post** | **Δ** | **p** | **r (rb)** |
| --- | --- | --- | --- | --- | --- | --- |
| Knowledge | High-risk only require reporting | 3.76 | 4.13 | 0.37 | 0.078 | 0.277 |
| Knowledge | OTC need no reporting | 4.05 | 4.28 | 0.23 | 0.068 | 0.321 |
| Knowledge | Only serious reactions require reporting | 3.58 | 4.05 | 0.47 | 0.011 | 0.434 |
| Knowledge | Physician is solely responsible | 4.21 | 4.37 | 0.17 | 0.200 | 0.227 |
| Perception | System is clear for providers | 3.68 | 4.19 | 0.51 | 0.002 | 0.508 |
| Perception | System is easy to access | 3.72 | 4.14 | 0.42 | 0.004 | 0.435 |
| Perception | Reporting supports patient safety | 4.31 | 4.44 | 0.13 | 0.473 | 0.134 |
| Barriers | No easy reporting system | 3.29 | 3.31 | 0.01 | 0.804 | 0.042 |
| Barriers | Case not important enough | 3.58 | 3.04 | -0.54 | 0.003 | -0.487 |
| Barriers | Unsupportive culture | 3.45 | 3.38 | -0.06 | 0.677 | -0.067 |
| Barriers | Workload | 3.38 | 3.41 | 0.03 | 0.875 | 0.026 |
| Barriers | Lack of knowledge | 3.15 | 3.22 | 0.06 | 0.671 | 0.068 |
| Barriers | Fear of consequences | 2.58 | 2.82 | 0.24 | 0.152 | 0.225 |
| Barriers | No feedback | 3.05 | 3.12 | 0.06 | 0.870 | 0.028 |
| Barriers | No incentives | 3.01 | 3.08 | 0.06 | 0.475 | 0.116 |
| Facilitators | Educational brochures | 4.04 | 4.08 | 0.04 | 0.588 | 0.088 |
| Facilitators | Practical training | 4.10 | 4.14 | 0.04 | 0.627 | 0.081 |
| Facilitators | Recognition or incentives | 3.90 | 4.01 | 0.12 | 0.448 | 0.134 |
| Facilitators | Digital solutions | 4.05 | 4.14 | 0.09 | 0.444 | 0.125 |
| Facilitators | Feedback after reporting | 4.01 | 4.01 | 0.00 | 0.922 | -0.019 |
| Facilitators | Dedicated support staff | 4.16 | 4.12 | -0.04 | 0.818 | -0.041 |

*Barrier items are scored in their original direction (higher = greater barrier); no barrier item is reverse-keyed. r (rb), matched-pairs rank-biserial correlation. Three items had N=77 from pairwise deletion of missing values.*

**Supplementary Table S2. Gender differences in baseline construct scores (Mann-Whitney U test; N male=55, N female=23).**

| **Construct** | **Male M(SD)** | **Female M(SD)** | **U** | **p** |
| --- | --- | --- | --- | --- |
| **Knowledge** | **3.77 (0.76)** | **4.20 (0.65)** | **838.0** | **0.024** |
| **Perception** | **3.90 (1.01)** | **3.91 (0.87)** | **616.5** | **0.863** |
| **Barriers** | **3.14 (0.36)** | **3.30 (0.28)** | **717.0** | **0.356** |
| **Facilitators** | **4.05 (0.75)** | **3.97 (0.95)** | **658.5** | **0.774** |

**Supplementary Table S3. Spearman Correlations Between Baseline Construct Scores and Age or Years of Experience**

| **Construct** | **Variable** | **Spearman r** | **p-value** |
| --- | --- | --- | --- |
| Knowledge | Age | -0.166 | 0.146 |
| Knowledge | Years of Experience | -0.119 | 0.298 |
| Perception | Age | -0.190 | 0.095 |
| Perception | Years of Experience | -0.162 | 0.155 |
| Barriers | Age | 0.099 | 0.388 |
| Barriers | Years of Experience | 0.105 | 0.359 |
| Facilitators | Age | -0.041 | 0.719 |
| Facilitators | Years of Experience | -0.072 | 0.529 |

**Supplementary Table S4. Professional-Level Differences in Baseline Construct Scores (Kruskal-Wallis H Test)**

| **Construct** | **H Statistic** | **df** | **p-value** |
| --- | --- | --- | --- |
| Knowledge | 0.923 | 4 | 0.921 |
| Perception | 7.263 | 4 | 0.123 |
| Barriers | 7.111 | 4 | 0.130 |
| Facilitators | 0.744 | 4 | 0.946 |

**Supplementary Table S5. Discriminant Validity — Inter-Construct Spearman Correlation Matrix (Pre-Intervention, N=78)**

| **Construct** | **Knowledge** | **Perception** | **Barriers** | **Facilitators** |
| --- | --- | --- | --- | --- |
| Knowledge | — | 0.095 | -0.199 | 0.206 |
| Perception | 0.095 | — | -0.346 | 0.371 |
| Barriers | -0.030 | -0.135 | - | 0.239 |
| Facilitators | 0.206 | 0.371 | -0.055 | — |

**Supplementary Table S6. Convergent validity: mean inter-item correlation (MIIC) by construct and phase.**

| **Construct** | **MIIC pre** | **MIIC post** | **At or above 0.20 threshold** |
| --- | --- | --- | --- |
| Knowledge | 0.307 | 0.526 | Yes |
| Perception | 0.739 | 0.825 | Yes |
| Barriers | -0.043 | -0.033 | No (formative index, as expected) |
| Facilitators | 0.723 | 0.860 | Yes |

**Supplementary Table S7. Provider education and community-outreach materials.**

| **Type** | **Title or content** | **Format** | **Aim** |
| --- | --- | --- | --- |
| Lecture | Basics of Pharmacovigilance | Live, 2 h | Build understanding of PV importance and the regulatory role |
| Course | Basics of Pharmacovigilance (13 units) | Self-paced | Set minimum standards for reporting quality and support safe practice |
| Posters (8) | ADR warning signs; QR-to-Tiqath journey; scan and report in 60 s; and others | WhatsApp | Clarify drug-safety concepts and guide digital ADR reporting |
| Videos (7) | QR scan to regulatory action; what is PV; side effect to signal; and others | WhatsApp | Strengthen medication-safety understanding and correct reporting |
| Podcast | Severity versus seriousness | 4:39 | Resolve a common concept confusion |
| Booklets (2) | QR scan to regulatory action | WhatsApp | Educate on drug safety and ADR reporting |

**Supplementary S8. STROBE Checklist**

| **#** | **Item** | **Location** | **Status** |
| --- | --- | --- | --- |
| 1a | Title/Abstract: indicate design | Title; Abstract Methods | Addressed |
| 1b | Informative/balanced summary | Abstract | Addressed |
| 2 | Background/rationale | Introduction §1–5 | Addressed |
| 3 | Objectives | Introduction, final paragraph | Addressed |
| 4 | Study design | Methods, Study Design and Setting | Addressed |
| 5 | Setting | Methods, Study Design/Intervention | Addressed |
| 6a | Eligibility criteria | Methods, Participants and Eligibility | Addressed |
| 6b | Matching criteria | — | Not applicable — no matched groups |
| 7 | Variables defined | Methods, Data Collection Instrument | Addressed |
| 8 | Data sources/measurement | Methods, Data Collection Instrument | Addressed |
| 9 | Bias | Discussion, Limitations | Addressed |
| 10 | Study size | Methods, Sample Size | Addressed |
| 11 | Quantitative variables | Methods, Statistical Analysis | Addressed |
| 12a | Statistical methods | Methods, Statistical Analysis | Addressed |
| 12b | Subgroup methods | Methods, Statistical Analysis | Addressed |
| 12c | Missing data handling | — | Not reported in the manuscript |
| 12d | Loss to follow-up | — | Not applicable |
| 12e | Sensitivity analyses | Methods, Statistical Analysis | Addressed |
| 13a | Participant numbers | Results, Participant Characteristics | Addressed |
| 13b | Non-participation reasons | — | Not reported in the manuscript |
| 13c | Flow diagram | — | Not applicable |
| 14a | Participant characteristics | Results; Table 1 | Addressed |
| 14b | Missing data counts | — | Not reported in the manuscript |
| 14c | Follow-up time | Methods, ADR Reporting Data Collection | Addressed |
| 15 | Outcome data | Results; Tables 2–3; Figure 2 | Addressed |
| 16a | Main results | Results, Table 3 | Addressed |
| 16b | Category boundaries | — | Not applicable |
| 16c | Absolute risk | — | Not applicable |
| 17 | Other analyses | Results, Subgroup Analyses | Addressed |
| 18 | Key results | Discussion §1 | Addressed |
| 19 | Limitations | Discussion, Limitations | Addressed |
| 20 | Interpretation | Discussion §2–6; Conclusion | Addressed |
| 21 | Generalisability | Discussion, Limitations | Addressed |
| 22 | Funding | Declarations, Funding | Addressed |

*Reporting guideline: STROBE, applied to this pragmatic pre–post real-world evaluation.*

**Supplementary S9 — Ethics Approval Documentation**

**Supplementary S10 — Full Bilingual Questionnaire**
